# In-line three-dimensional holography of nanocrystalline objects at atomic resolution

**DOI:** 10.1038/ncomms10603

**Published:** 2016-02-18

**Authors:** F.-R. Chen, D. Van Dyck, C. Kisielowski

**Affiliations:** 1 Department of Engineering and System Science, National Tsing-Hua University, 101 Kuang-Fu Road, Hsin Chu 300, Taiwan; 2 EMAT, Department of Physics, University of Antwerp, 2020 Antwerpen, Belgium; 3 Lawrence Berkeley National Laboratory, The Molecular Foundry and Joint Center for Artificial Photosynthesis, One Cyclotron Road, Berkeley California 94720 USA

## Abstract

Resolution and sensitivity of the latest generation aberration-corrected transmission electron microscopes allow the vast majority of single atoms to be imaged with sub-Ångstrom resolution and their locations determined in an image plane with a precision that exceeds the 1.9-pm wavelength of 300 kV electrons. Such unprecedented performance allows expansion of electron microscopic investigations with atomic resolution into the third dimension. Here we report a general tomographic method to recover the three-dimensional shape of a crystalline particle from high-resolution images of a single projection without the need for sample rotation. The method is compatible with low dose rate electron microscopy, which improves on signal quality, while minimizing electron beam-induced structure modifications even for small particles or surfaces. We apply it to germanium, gold and magnesium oxide particles, and achieve a depth resolution of 1–2 Å, which is smaller than inter-atomic distances.

## Abstract (web summary)

The resolution of transmission electron microscopes allows the imaging of single atoms and determination of their locations in a plane. Here, the authors present a tomographic method to recover the three-dimensional shape of a crystalline particle without the need for sample rotation.

## Graphical abstract

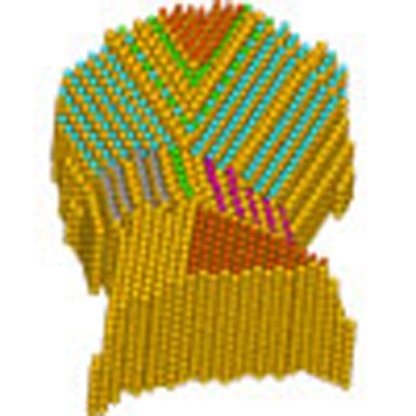

In the late 1950s Richard Feynman pointed out^1^ that ‘It would be very easy to make an analysis of any complicated chemical substance; all one would have to do would be to look at it and see where the atoms are.' In principle, the latest generation aberration-corrected transmission electron mocroscopes can achieve this goal^2^,^3^,^4^ but for a variety of reasons one is still far away from a reliable method that would meet Feynman's challenge of extracting the three-dimensional (3D) position of all the atoms in an object, to understand its physical and chemical properties^5^,^6^,^7^,^8^,^9^,^10^,^11^,^12^,^13^. A most noticeable bottleneck is the large accumulated electron dose required to produce tilt series of atomic resolution images, because electron dose rates are commonly chosen large (10^4^–10^5^ eÅ^−2^ s^−1^) to achieve a needed resolution around 1 Å and single atom sensitivity. Any such single image can exhibit uncontrolled electron beam-induced surfaces alterations or even bulk modifications, in particular if particles are small^14^,^15^,^16^,^17^. Therefore, only a few favourable cases allowed for an extraction of atom positions in the beam (*z*) direction with high precision. They included the study of a graphene double layer^18^ that can tolerate extraordinary large electron dose rates, tomography of embedded nanocrystals where a sacrificial matrix protects the nanoparticles^19^ or a 3D structure determination by comparing experimental images with theoretical expectations that typically include assumptions of debatable validity^20^,^21^. On the other hand, small crystalline particles are known to exhibit drastically altered bulk or surface properties such as their catalytic activity. Consequently, there is a strong need for a tomographic technique with atomic resolution that can maintain the pristine structure of small objects, which requires imaging with small electron dose rates.

In this study we present a self-consistent approach to recover the 3D atomic structure of nanocrystalline particles from single projections by exploiting the dynamic nature of electron scattering and pursuing a quantitative interpretation of the electron exit wave reconstructed from focal series of high-resolution images. The exit wave contains not just the usual intensity, but the entire field information, amplitude and phase, which is the same as ‘ holography'. In particular, this reconstruction method allows capturing images with choosable dose rates that can be adjusted to maintain structural integrity during the imaging process without compromising spatial atomic resolution and single atom sensitivity^22^,^23^,^24^,^25^,^26^. Currently, exit waves can be reconstructed from images captured with dose rates reduced to a level of roughly 1 atto-Ampere per Å (6 eÅ^−2^ s^−1^). Moreover, it is pointed out that the reconstructed electron wave in an image plane is not identical to the wave at the exit surface of the crystal, because crystals exhibit a shape and surfaces are often not flat at the atomic scale but exhibit roughness. Therefore, focus values with respect to a common image plane change locally.

If crystalline objects are imaged along a zone axis orientation in an electron microscope, the incident electrons are trapped in the strong electrostatic potential of the atomic columns in beam direction. This trapping of electrons is commonly described by electron channelling^27^,^28^, which has all ingredients for a full 3D quantification of image contrast, as the scattering process critically depends on column length and its chemical composition but only weakly on electron channelling in neighbouring columns as long as samples are thin and the column spacing exceeds ∼1 Å in the image plane. Therefore, the exit wave of a crystalline object in zone orientation can be analysed column by column. Within each column, successive atoms are aligned with an equidistant spacing set by the crystal lattice and they act as lenses that focus and defocus the propagating electron wave periodically with increasing column length. Thus, the electron wave inside the column oscillates periodically with sample thickness and contains depth information. Element-specific contrast changes can be observed in high-performance microscopes, because atoms are discrete objects with a characteristic scattering power yielding element-specific phase changes. Our procedure to extract quantitative information from exit waves is described in the Methods section and partly in previous publications^18^,^23^. It addresses the challenge how to extract for every column its mass and its distance to a common reference plane with single-atom sensitivity and a precision in beam direction that exceeds interatomic distances. Once focus and mass of all atomic columns are deduced from a single-projected exit wave, the 3D structure of the crystalline particle can be reconstructed. Conveniently, atomicity provides an internal and self-consistent calibration standard that can be used to recover any sample shape from only one projection at truly atomic resolution. In addition, it is shown for exit waves recorded with the TEAM 0.5 microscope^24^ how a chosen electron dose rate affects the absolute phase values for the detection single atoms. In summary, we show analysing electron channelling along atom columns allows for a full 3D quantification of the atomic structure as long as the atom columns are homogeneously occupied. Concerning defects, our method is readily available to investigate edge-on dislocations or planar defects such as twin boundaries in 3D, which will be demonstrated later in an experimental exit wave of a gold particle. A point defect such as a vacancy can be detected as an atomic step in the surface with respect to the neighbouring columns if single projections are used.

## Results

### Mass and focus circles for 3D reconstruction

**Figure 1:**
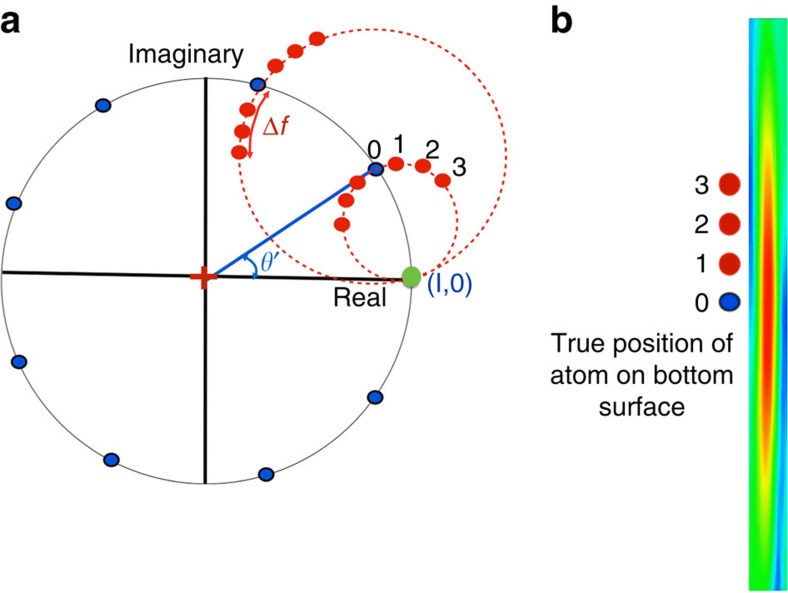
Schematic representation of the exit wave function in Argand space and propagation intensity. (**a**) The complex values of pixels in the centre of each atomic column are represented as blue dots. The red dots correspond to pixels of the wave function if propagated across a distance Δ*f* away from the exit surface of the sample. Black circle is called ‘mass circle' and the red dashed circle is called ‘defocus circle'. (**b**) The propagation intensity of one atomic column. The true position of atom at the bottom exit surface is at the position of maximum intensity (blue point).

The exit wave Ψ_e_(**r**, **z**), at a particular image plane, can be expressed analytically as^23^:


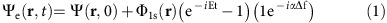


where *t* is the mass thickness of the sample, Ψ(**r**,0) is the incident wave, Φ_1s_(**r**) is the 1s eigenstate of the projected electrostatic potential of the atom column with eigenenergy E. *α* is a constant and Δ*f* is the focus difference given by the distance of the image plane to the exact exit surface defined by the last atom in a column. The exit wave is complex and can be represented in an Argand plot. Figure 1a shows the Argand plot of pixels from the centre of atom columns. It is clear that the two factors (*e*^−*i*Et^−1) and (1−e^−*iα*Δ*f*^) from equation describe two circles called ‘mass (Et) circle' (black circle) and ‘defocus (*α*Δ*f*) circle' (red circles), respectively. It is seen that information about the column mass and its local focus are orthogonal. If the sensitivity of a microscope suffices to isolate the contrast contribution from scattering at single atoms, the ‘mass' values will be discrete and their regular spacing will give an ultimate mass calibration. Similarly, discrete focus values must be detected if the spatial resolution in beam direction is smaller than the periodic distance between successive atoms in a column.

The Argand plot (Fig. 1a) explains in a natural manner how shape changes can be separated from column thicknesses (masses) by applying local wave propagation. The blue points on the black mass circle represent columns of different thickness characterized by the angle *θ*' between any blue point and the incident (or vacuum) wave at (1,0). If the column mass increases, *θ*' increases proportional to the projected thickness (mass) of the column. To convert the column mass *θ*' into the number of atoms in a column, the phase *θ*' must be divided by the phase change per single atom *θ*, which roughly increases with the atomic number *Z*^2/3^ (ref. 26) as used in the Methods and Table 1. Experimentally, however, the scattering power of atoms measured by *θ* is not a constant, because it is modulated by thermal vibrations and by electron beam-induced sample excitations^26^. Nevertheless, the complex wave values located directly on the mass circle represent columns where the exit surface of the sample coincides with an image plane so that there is no focus difference between them. However, shape or surface roughness at the bottom of a sample must locally create finite focus deviations, because the altered geometry moves away the exit wave from the image plane along the red defocus circle. The arc length between the red and blue dots in Fig. 1a is a measurement of this local defocus and wave propagation must be used to refocus all atomic columns into the same image plane for a quantitative analysis. It is worth noting that the intensity of the exit wave always decreases as the electron wave is propagated away from the common image plane (Fig. 1b). We use the criterion of maximum propagation intensity (MPI) to determine local shape changes at the bottom of the sample by measuring the local defocus values and further refine them with the Big-Bang scheme^18^ to a nominal precision of better than 1 Å. Once column thickness and surface shape at the bottom of the sample are known, their linear combination creates a tomogram. The detailed procedures and analyses are given in Methods.

### Analysis of experimental exit wave functions

**Figure 2:**
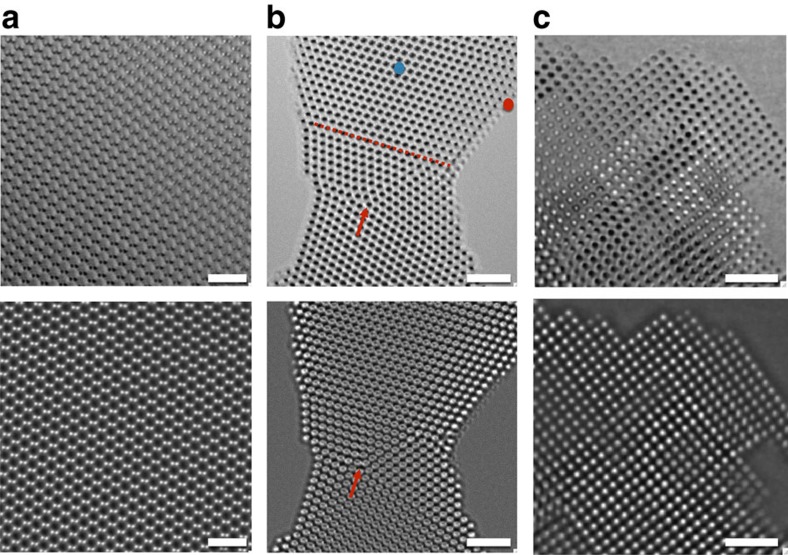
In-line holograms acceleration voltage=300 kV. Dose rates are given in Table 1. (**a**) Ge[110] and (**b**) Au[110] were reconstructed from focal series of 35 images with a focus step of 2 nm and Cs=0 mm. (**c**) MgO[100] was reconstructed from focal series of 80 images with a focus step of 1 nm and Cs=−0.015 mm. Top and bottom rows show the amplitude and the phase of the reconstructed electron exit wave functions, respectively. Scale bar, 1 nm in all cases. It is noteworthy that there are edge-on twin boundaries present in the imaged gold sample (**b**, one is highlighted by a dash line). At the intersection of three twin boundaries, a present end-on dislocation marked by an arrow. In addition, in phase image of **b**, many atom columns assume ‘donut' shapes because mass and focus information are convoluted.

**Figure 3:**
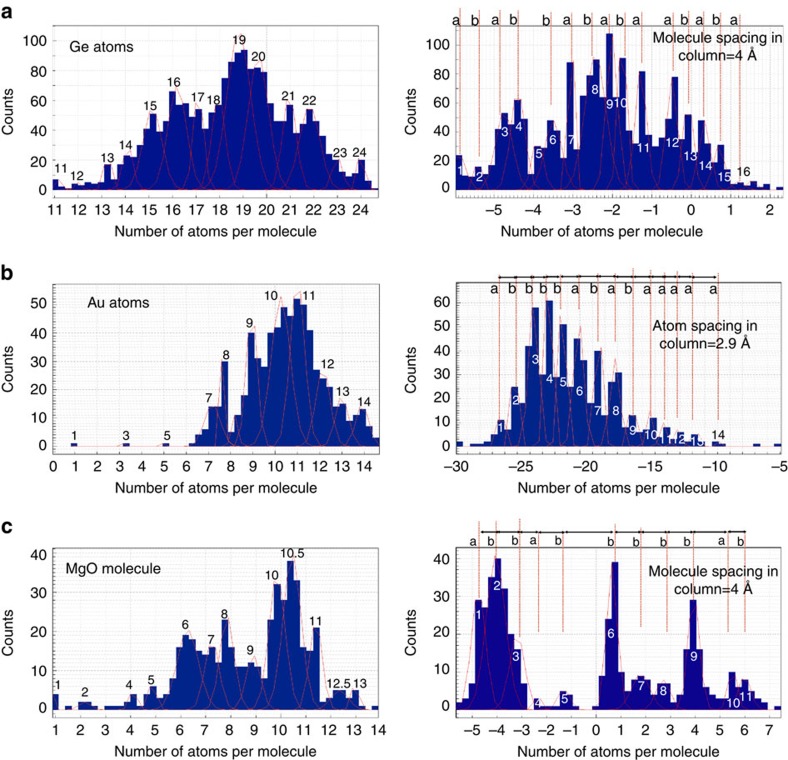
Column mass and focus histograms. (**a**) Germanium and (**b**) gold values are given in terms of single atoms. (**c**) MgO graphs refer to single molecules. Gaussian functions (red lines) are fitted to their width, which is given in form of an averaged 2*σ error bar in Table 1. In focus graphs, the number of atoms/molecules are converted into focus values by multiplication with their listed spacing in beam direction. For germanium and gold, **a** and **b** sites refer to the existing (110) surface corrugation. In case of MgO [100], a surface corrugation is absent but surfaces are either terminated by Mg or by O atoms.

Intentionally, we prepared a semiconductor, a metal and a ceramic sample by different techniques, namely by ion milling, thin film deposition and by electron beam-assisted processing, to obtain differently shaped objects. Amplitude and phase of their exit wave are shown in Fig. 2. Figure 2a displays a Ge [110] sample prepared from a bulk Ge single crystal by mechanical polishing and successive ion milling^29^, and one expects the creation of surface steps forming a wedge-shaped sample with shallow angles. The gold [110] sample was grown by physical vapour deposition on germanium^30^. After growth, the Ge substrate was etched away creating a free-standing metal sample with flat bottom and rounded top (Fig. 2b). It is emphasized that in this case twin boundaries and a dislocation core are included in the analysis to make the point that extended defects can be analysed by our procedure. Finally, the MgO sample originates from a polished MgO [001] single crystal, which was prepared in [100] cross-section^31^ and exposed to the high brightness electron beam at 300 kV for several minutes. *In-situ* observations revealed that the high-energy electron beam removes all sample preparation induced surface roughness and forces the formation of the stacked cube structure with one global [100] zone axis orientation (Fig. 2c). All images of Fig. 2 show crystals are suspended in the high vacuum of the electron microscope and the support films are not visible in the field of view. Moreover, we did not find any evidence for an attachment of residual gas molecules from the high column vacuum to the surfaces of the samples, as a cold N_2_ trap was used. The expected geometrical features including the presence of surface steps that, however, are not obvious in these images, except for contrast fluctuations in the amplitude image of the MgO [100] sample (Fig. 2c), which suggests the presence of MgO cubes. This information is simply masked by mixing sample shape with column length in the experimental images as described earlier. For their deconvolution we apply MPI and column mass measurements (Methods) to each atom column of the electron exit waves (Fig. 2), which provides the histograms of column mass and defocus values (Fig. 3). It is seen that all histograms reveal discrete sets of peaks, which are periodically spaced. Image simulations confirm that the incremental steps between adjacent peaks correspond to the addition of single atoms or molecules to atom columns with a periodic spacing in the beam direction set by the crystal structures of the materials. In a second process, we determine the confidence levels of these measurements by fitting Gaussians to the accumulation points (Fig. 3). This allows for an extraction of error bars that are given as 64.2% probability values (2*σ* values) of the measurements and are listed in Table 1.

**Figure 4:**
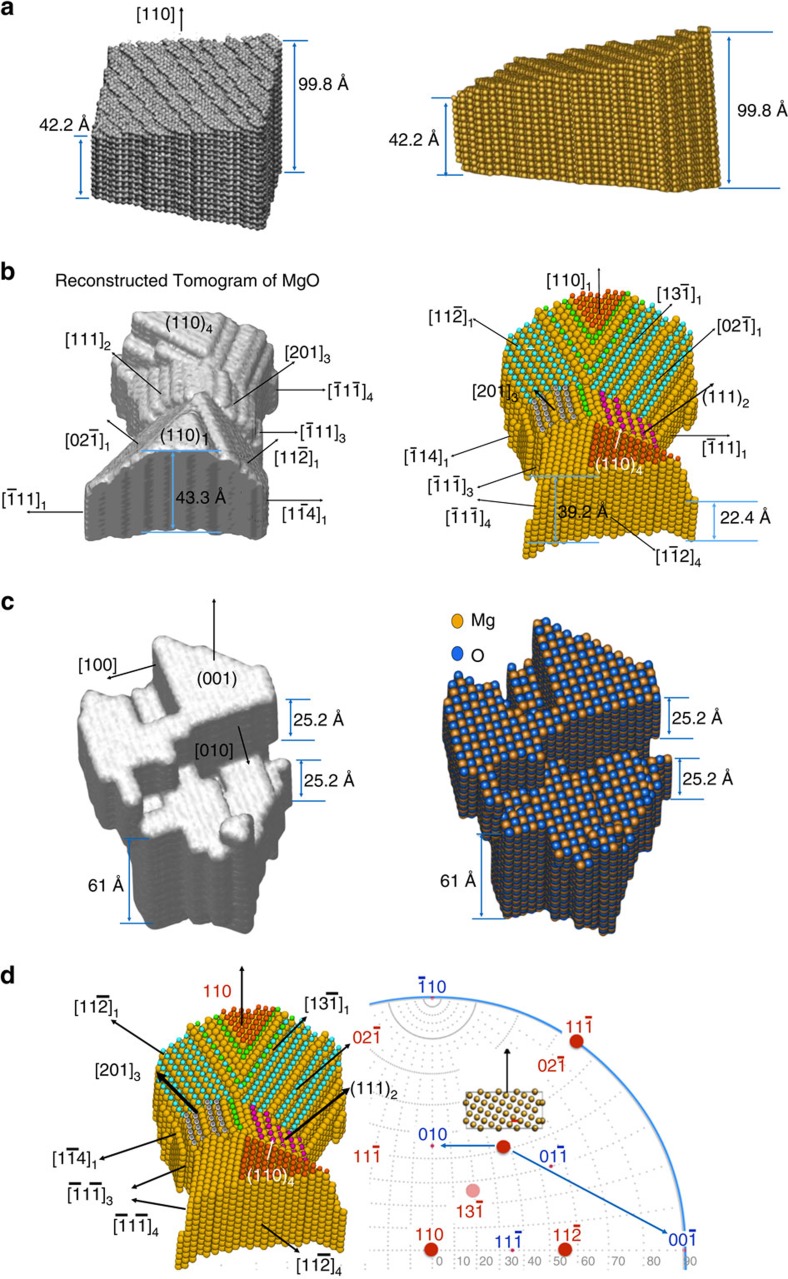
Atomic resolution tomograms. (**a**) Surface shape and atomic structure views of the Ge [110] sample. (**b**) Surface shape and atomic structure views of the Au[110] sample. The facets are highlighted with different colours. (**c**) Surface shape and atomic structure views of the MgO [100] sample. Orange atoms: Mg, blue atoms: O (the size of the atoms is intentionally enlarged to render the shape of the particle). (**d**) The Wulf net shows the relationship of the high-energy facets (red dots) with low indexed facets for four grains indexed with *i*=1… 4. In grain 1, a [02] surface facet can be formed by low energetic [010] and [00] (red symbols) surfaces as shown by the insets and observed in the tomogram.

To convert column mass values into radians, we determined experimentally the phase changes of the electron wave caused by scattering at one gold atom and one MgO molecule (Methods). Table 1 lists these phase values. It is seen that the phase of the exit wave is changed by 0.21±0.07 rad by passing through a single gold atom in an atomic column or by 0.08±0.02 rad if it is passing through a single MgO molecule. A value of 0.11 rad for scattering at one Ge atom is estimated using a *Z*^2/3^ dependence. Remarkably, it is also seen that error bars increase with increasing dose rates, suggesting that measurements with a best element differentiation can only be performed if dose rates are kept low. In addition, column locations in the beam direction can be determined to a precision 1.2–2.4 Å on an absolute scale. Consequently, depth resolution has reached interatomic distances in thickness reconstructions from single projections. It is now straightforward to create 3D tomograms from these measurements, as the focus values describe the exit surface profile of the sample and the column length is given by the number of atoms of known spacing along the column length. In this manner, we have created the tomograms (Fig. 4) that show all geometrical properties that one expects to be imprinted by the chosen sample preparation procedure. The number of atoms in each tomogram is a time average that is dose rate dependent and equals 35,389 for Ge, 4,883 for Au and 10,750 (Mg, O) for MgO.

Specifically, the tomogram of Ge[110] in Fig. 4a shows a wedge-shaped sample prepared by ion milling with a low incidence angle (∼6°). Usually such wedges are formed by irregularly spaced terraces on both sides of the sample^31^, which is confirmed by this tomogram. The Au [110] sample is dome shaped with a reasonably flat bottom at the side of the crystal initially attached to the germanium substrate. In addition, pronounced facets and surface reconstructions are seen. It was established^32^ that high beam currents rapidly alter all surfaces of gold crystals during the acquisition of high-resolution images. The process transforms the material into a thermodynamically more stable form that can be recognized by the exposure of (111) surfaces. Such atom rearrangements are driven by the low surface energy *γ*_Au_(111)^33^ and are visible in Fig. 4b. Consequently, the tomogram depicts a crystal structure averaged over the 60 seconds acquisition of the focus series, while the surfaces of the crystal were altered from image frame to image frame. The misleading impression that a static situation would be considered only exists, because surface diffusion is fast compared with the image acquisition time^34^ and the loss of single atoms from atom columns is hard to detect. The MgO [100] particle does not exhibit the typical wedge shape that is characteristic for sample initially prepared by ion milling. Instead, a shape transformation took place into the stack of cubes shown in the tomogram of Fig. 4c during the prolonged exposure of the sample to a high electron beam current of ∼50,000 eÅ^−2^ s^−1^ before the experiment. The focus series itself was recorded with a reduced beam current of 1,300 eÅ^−2^ s^−1^. Similar to the Au sample, an exposure of the material to high beam currents triggers the formation of a thermodynamically more stable shape that, however, creates cubes with exposed (100) surfaces in MgO, because the surface energy *γ*_MgO_(100) is the lowest^35^. From the tomogram, it is seen that the edge length of such cubes varies between 2.5 and 6 nm. The sample thickness at the highlighted vacuum/MgO interface exceeds 6 nm and any {100}/vacuum interface is atomically flat.

## Discussion

Successful reconstructions of sample shapes from single projections date back to the early 1990s (ref. 11) when the shape of a silicon crystal was reconstructed from a single high-resolution image. However, this approach did not allow to identify single atoms or surface steps, because lens aberrations were ignored and a procedure was lacking to separate column length from surface profile. Ever since, progress was steady^12^,^18^,^19^,^21^,^23^ and has now reached the point that atom locations can indeed be determined in 3D so that Feynman's challenge can be met. Instead, debates evolve around the implementation of most suitable methods, the validity of recovered values, the necessity of recording many projections and the need to control of beam–sample interactions.

The development of exit wave reconstruction methods is a key element for the achieved progress, as it allows to describe the dynamic electron channelling in an Argand plot, which is transparent and anchored in physics^36^,^37^,^38^,^39^. The Argand plot (Fig. 1) explains in a natural manner how the vertical position of a column can be separated from the column mass by applying local wave propagation and how the column mass can be quantified. It also allows to understand how point defects affect the mass of a column. A vacancy, for example, will reduce the column mass by a single atomic step that can be predicted and measured if the related phase change/atom exceeds the noise level. Certainly, grain boundaries and dislocation cores can be included in the analysis (Fig. 2b). Moreover, our tomograms (Fig. 4) show that the thickness of the analysed crystalline objects exceeds now 10 nm, which makes the tool generally applicable for investigations of nanocrystals and catalysts. In general, the sample thickness is limited to a full oscillation period (or extinction distance) of the channelling electron wave in the order of tens of nanometres. It is noted that beside electron microscopy, 3D atom probe (3D-AP) is also available for atom counting^40^. Two complementary differences between 3D-AP technique are that our technique accounts for every atom, while ∼50% of all atoms can escape an AP observation and AP can detect much smaller impurity levels.

The measured phase change per Au atom and per MgO molecule (Table 1) of 0.21±0.07 and 0.08±0.02 rad, respectively, can directly be compared with multislice calculations using the electron scattering factors by Doyle and Turner^41^, and a reasonable Debye–Waller factor of 0.5 Å^2^, which accounts for a Gaussian distribution of averaged atom displacement by ∼8 pm (ref. 41). We calculate an expected phase change of 0.40 rad for a gold atom and 0.09 rad for a MgO molecule (Table 1). By comparison, these theoretical expectations exceed the measurements by factors of 2.5 and 1.1, and the larger discrepancy occurs for the Au atoms where the images were recorded with the largest electron dose rate of 45,000 eÅ^−2^ s^−1^ (Table 1).

**Figure 5:**
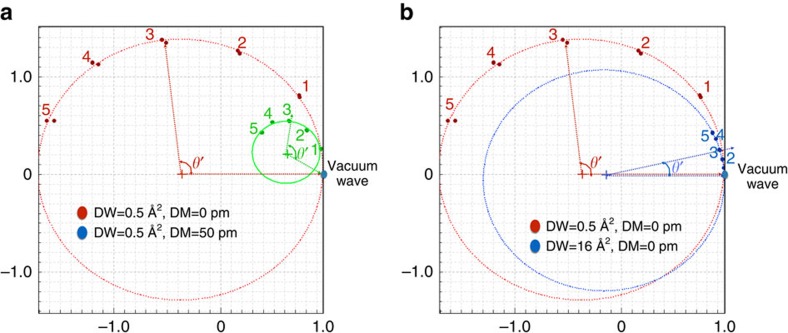
Effects of mechanical damping and potential softening to Argand plots. (**a**) Red Argand plot shows exit waves reconstructed from simulated images of Au [001] crystal with Debye–Waller (DW) factor of 0.5 Å^2^ with no mechanical damping (DM), whereas the green Argand plot is obtained from the same crystal but with mechanical damping (DM=50 pm) of the contrast transfer function. The numbers in the plot correspond to number of atom. (**b**) In our case, phases are reduced due to reversible electron beam-induced object excitations in the image formation process. This electron beam stimulation effect is modelled as a higher Debye–Waller factor of 16 Å^2^ with DM=0 pm.

It is instructive to investigate the impact of different damping functions on the signal strength using an Argand plot. Contribution from damping functions such as a poor modulation transfer function of a camera or mechanical vibrations, for example, simply reduce the diameter of the Argand circle but do not affect the phase change per atom as long as phase changes are measured from the centre of an Argand circle (Fig. 5a). The exit wave for the red Argand plot (Fig. 5a) is reconstructed from simulated images of a Au [001] crystal with a Debye–Waller factor of 0.5 Å^2^, whereas the green Argand plot is obtained with the same parameter set, except for an additional mechanical damping of the contrast transfer function by 50 pm, which coincides with the information limit resolution of TEAM 0.5. It is seen that the phase change per atom is maintained if measured from the origin of the Argand circle even though its diameter is largely reduced. Consequently, we do not correct for a poor camera performance or mechanical vibrations, because such corrections only boost high-frequency noise but leave phases unaffected if described in an Argand plot. Instead, we fit Argand circles to the data points and translate the origin of the circle to (0,0). On the other hand, damping processes such as electron beam-induced atom vibrations can soften the scattering potential and reduce the phase values for scattering at single atoms. If we model electron beam-induced object excitations of 45 pm by using a larger Debye–Waller factor of 16 Å^2^=8*π*^2^(45)  pm^2^ (ref. 41), the phases are greatly reduced, which leads to the blue circle in Fig. 5b. This description is consistent with the view that reversible electron beam-induced object excitations contribute to the image formation process. As such excitations can cause large displacements and decrease logarithmically with decreasing dose rates^26^, low dose rate electron microscope becomes advantageous or even mandatory if it is needed to maintain the pristine structure of small particles^42^, surfaces or even molecules^43^.

**Figure 6:**
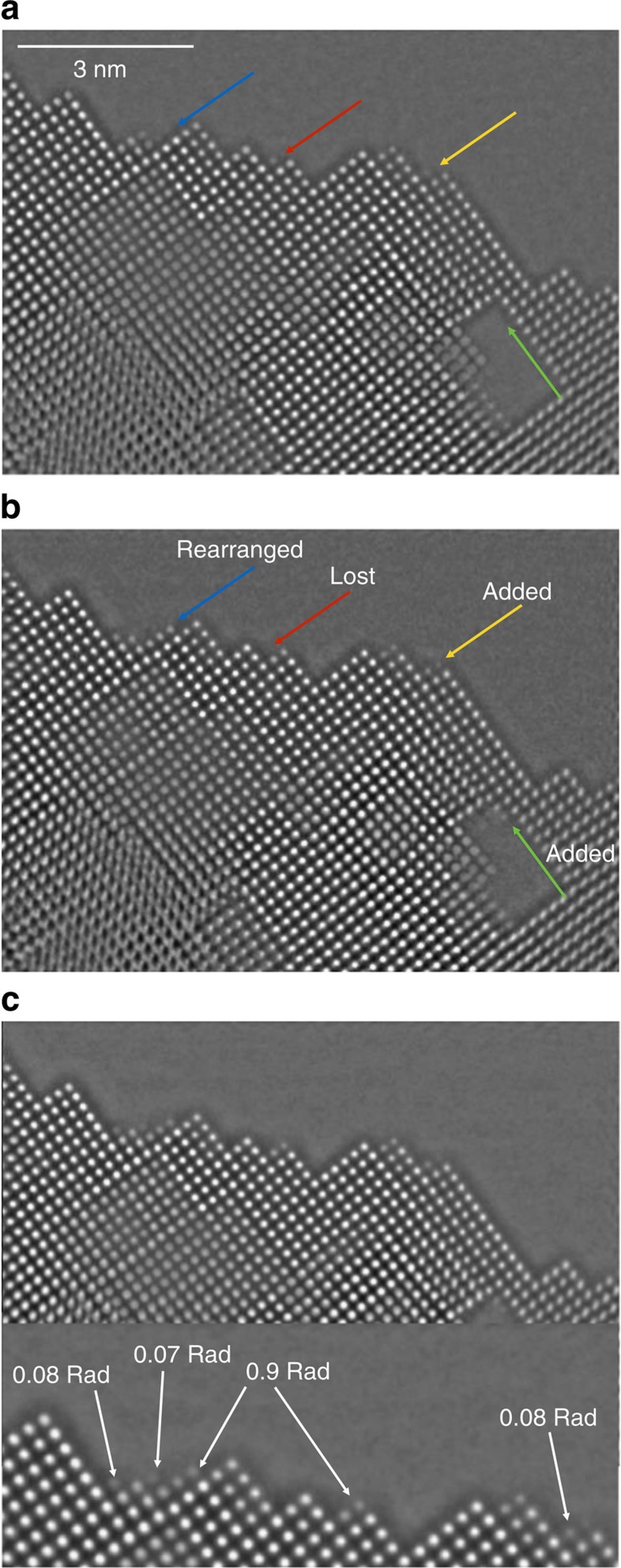
Reconstructed exit waves from different time series. (**a**) Reconstructed exit wave from the first 35 time series images. (**b**) Reconstructed exit waves from the 35th to the 70th images. The edge atoms are obviously altered via electron beam–sample interaction. (**c**) Reconstructed exit waves using the images of whole time series. The phase value from the edge atoms can be read to be ∼0.08 rad. Scale bar, 3 nm in all cases.

To reduce electron beam-induced sample alterations, electron dose rates were dropped to 1,300 eÅ^−2^ s^−1^ for the acquisition of the exit wave of MgO. It is advantageous that focal series of images were recorded, because they can be used to study and track electron beam-induced object changes by splitting the data set into different subsets that can be reconstructed separately. For example, Fig. 6a is a reconstruction from the first 35 images of the MgO data set, Fig. 6b is reconstructed from images 36 through 70 and Fig. 6c makes use of the entire focus series. Arrows in Fig. 6a,b mark locations where the electron beam visible changed the sample/vacuum interface. An estimated 5–10% of the all surface sites are affected by the chosen dose rate and one has to assume that such alterations also occur on the top and bottom of the sample where they escape a direct observation. In fact, it was recently reported that structural integrity of oxide catalysts smaller than 5 nm can only be maintained if dose rates are kept well below 1,000 eÅ^−2^ s^−1^ (ref. 44). It is also seen in Fig. 6 that the displaced entities on the MgO [100] surfaces occupy regular lattice sites and do not distort the MgO lattice if attached somewhere else. Therefore, it is compelling that they are elements of the crystal structure. At the vacuum/crystal interface, we commonly measure a contrast change of 0.07–0.09 rad (Fig. 6c). Smaller phase changes around 0.04 rad could be detected but occur rarely. If the contrast change of 0.04 rad is assigned to single Mg or O atoms, the most commonly occurring phase change of 0.08 rad represents the presence of MgO molecules aligned in beam direction. In fact, a phase value of 0.08 rad is very close to the calculated phase change of 0.09 rad for a molecule (Table 1). The common occurrence of the molecular unit also explains the discrete nature of the histogram in Fig. 2c. Therefore, unlike the case of Ge[110] and Au[110], MgO molecules are the basic unit that differentiates column masses and entire MgO molecules are commonly displaced by the electron beam or added to different sites of the crystal structure. In addition, the calibration of the mass phase is consistent with a contrast dependence proportional to *Z*^2/3^ (refs 25, 26). In addition, it seems energetically unfavourable to attach single Mg or O atoms to the [100] surfaces of the MgO compound, because the bonding is ionic and electric charge would occur locally on a crystal surface that is of minimal surface energy^5^ if neutral. If carbon contaminants were present, they would also modulate the column masses with values around 0.04 rad. The rare occurrence of such column mass modulations proves that the crystal surfaces remain free of carbon contamination. Therefore, we find that electron irradiation can be used to form perfect cubes from MgO samples initially prepared by ion milling and consequently exhibited a wedge shape similar to that of the Ge[110] crystal.

Jia *et al.*^21^ reports on the 3D reconstruction of an almost identical MgO [100] cube from a single image. In this work, a dose rate is estimated from the reported counts 3,500 (ref. 21), exposure time 0.5 s and the photons per electron conversion rate 2 photons per electron at 300 keV to be approximately between 50,000 and 100,000 eÅ^−2^ s^−1^, which roughly equals the total dose of our experiment. By comparison, our experiment spreads this dose over a recording time of 120 s so that the dose rate is two orders of magnitude lower. Otherwise, their experimental conditions are very similar but the conclusions differ. It is remarkable that the authors^21^ determine a sample thickness of only 1.5–2.0 nm from an area close to the vacuum/MgO interface that is similar to ours and postulate the presence of residual gas contaminants on the MgO[100] surfaces to match the image contrast quantitatively with multislice calculations. As our self-consistent approach neither recovers sample areas of such small thickness nor provides evidence for the presence of surface contaminations, it is likely to be that the existing differences relate to electron beam-induced sample excitations. They greatly affect absolute values in such a manner if experiments are directly compared with theory as described in ref. 21.

In our experiments, structural rearrangements occur on the gold sample surfaces from image to image^32^,^45^ due to the choice of a high dose rate for the recording. One outstanding drawback of using high beam currents is a greatly increased error bar on the determination of the phase changes (Table 1), which prohibits a reliable element differentiation. Nevertheless, meaningful information can be extracted for the mono-elemental gold crystal. As the contrast is high and single gold atoms can be distinguished, structural feature showing equilibrium configurations at atomic resolution become visible in the tomogram. The formation of equilibrium structures is driven by surface energies *γ* that increase in the order *γ*_Au_(111)<*γ*_Au_(100)<*γ*_Au_(110) for face-centred gold crystals^33^. In the tomogram of Fig. 4b, the existence of large (111) facets confirms this view. This line of argumentation can be extended by considering that any other surface that can be reconstructed by exposing combinations of low energetic surfaces. For example, a missing row reconstruction occurs, because it increases low energy contributions from (111) surfaces instead of simply exposing a high energetic (110) surface and combinations of (111) with (100) surfaces yield similar effects. In Fig. 4d, the pronounced exposure of a (02–1) surface is highlighted that is formed by a combination of two {100} surfaces. However, the existing equilibrium configurations are distorted by the dynamic surface alterations induced by the large beam current. Nonetheless, the result strengthens our claim that structure recovery from single projection has emerged as a robust tool to determine atom positions in 3D at atomic resolution, which addresses Feynman's challenge.

The possibility to perform inline 3D holography at very low dose rates provides a powerful new tool for *in-situ* observation of structural transformation dynamics in small particles. This is particularly clear from the observation of the MgO particle, as both the Ge and the MgO samples were prepared by ion milling to form a wedge-shaped sample with random terraces. From the reconstructed tomogram, however, one can see that the Ge sample has kept this form but the MgO sample was transformed into a cubic shape with cubic protrusions terminated by (100) terraces.

Our working hypothesis is that the illuminating electrons transfer kinetic energy to the atoms in the sample, which cause them to vibrate. Certainly, near head-on collisions displace the atoms; however, as argued in refs 24, 25, most of them are displaced in a metastable position from which they can return to their original site. When the dose rate is sufficiently small, the transferred energy can be dissipated before being accumulated and the average kinetic energy of the atoms stays below threshold limits. In that case, all atoms remain close to their original sites but the apparent Debye–Waller factor can be larger than expected from the thermal motion alone^24^,^25^,^26^. Thus, low dose rates still transform samples locally but in a stationary manner with an average structure that can still be observed in high resolution electron microscopy. In contrast, dose rates that exceed the threshold cause additional collisions before the atoms return to their equilibrium sites and cause permanent damage and eventually destroys the material.

Conversely, permanent radiation damage can be retarded by reducing the dose rate below a certain threshold, which keeps the sample in a stationary regime between creation and annihilation of atom displacements that can still be observed by high resolution electron microscopy. In the case of the MgO sample, the atoms are constantly moving under the irradiation so that the object goes through many different unstable states. However, at the end all the atoms move to stable (100) planes where they are bound in a much more stable position around which they then constantly vibrate.

We argue it is a general phenomenon that most of the surface atoms are more easily displaced than bulk atoms. Under the electron beam, nano-objects are constantly transformed going through several intermediate structures until the atoms are finally grouped in more stable planes where they can then stay in a kind of steady state. Thus, irrespective of the original shape of the sample, it will always evolve into a stable structure that is stationary in the electron beam. Only when the dose rate exceeds a certain threshold, the sample will be damaged irreversibly. This hypothesis holds a very promising method to create nanocrystalline objects with a well-controlled shape by choosing appropriate dose rates.

Our results demonstrate that arbitrary 3D structures of nanomaterials can be recovered up to the level of single atoms from only one projection if the crystal structure is known and the material is homogeneous. However, it is noted that the electron dose rate used for imaging is important in two aspects. First, modification of the sample take place that can be controlled and, second, it induces a softening of the scattering potential that leads to an underestimation of the mass in atomic column. The method allows investigating defects such as edge-on dislocation or planar defects such as stacking fault and grain boundaries. Certainly, the addition of two additional projections of the same sample make any assumptions obsolete and fully resolves the internal structure of any crystalline object^12^.

## Methods

### Procedures for 3D reconstruction

As a result of electron channelling in the atomic columns, the exit wave function consists of sharp peaks superimposed on a constant background. For crystalline materials, basically, the exit wave function can be analysed column-by-column. The procedure of 3D reconstruction can be subdivided into several steps as follows: (1) determination of the true ‘*z*' height (focus) of the exit surface of a column from an image plane with the maximum propagation intensity (MPI) criterion by wave propagation along ‘defocus circles' as shown in Fig. 1; (2) refining the ‘*z*' height using the Big-Bang scheme^18^; (3) correcting the focus of each column wave by back propagation, that is, propagating the red dot to the blue dot (focus-corrected wave, FCW), which is located on the ‘mass circle' as shown in Fig. 1; and (4) extracting the wave values in the ‘valleys' between the atomic columns (background or valley wave).

(5) The phase of the exit wave of a column can suffer from a phase shift caused by the mean inner potential of the crystal and by atom vibrations, which results in a phase offset that causes an error in the determination of the column mass. This can be corrected using peak to valley ratios by dividing its complex peak value with the complex value of its neighbouring valley wave for every column. The phase of this normalized wave *θ*' can then be used as input to determine the column mass.

(6) Fitting a mass circle to the normalized FCW (blue dots). The centre of the mass circle is displayed as a red cross in Fig. 1.

(7) Measuring the phase of the vacuum wave from the centre of the mass circle. Usually, the vacuum wave is very close to (1,0) as indicated as a green dot. The phase of the vacuum wave is used as a reference for zero mass.

(8) Measuring the phase of the normalized FCW (blue dot) from the centre of mass circle. The column mass is given by the phase angle *θ*' between the normalized FCW and the vacuum wave. See Fig. 1.

(9) The column mass in units of the number of atoms can be deduced by dividing the *θ*' by a standard phase change *θ* per one atom (column 2 and 3 in Table 1). The value of the phase change *θ* per atom (molecule) is sensitive to the electron dose rate and will be described in Methods.

### Determination of the defocus of a column

**Figure 7:**
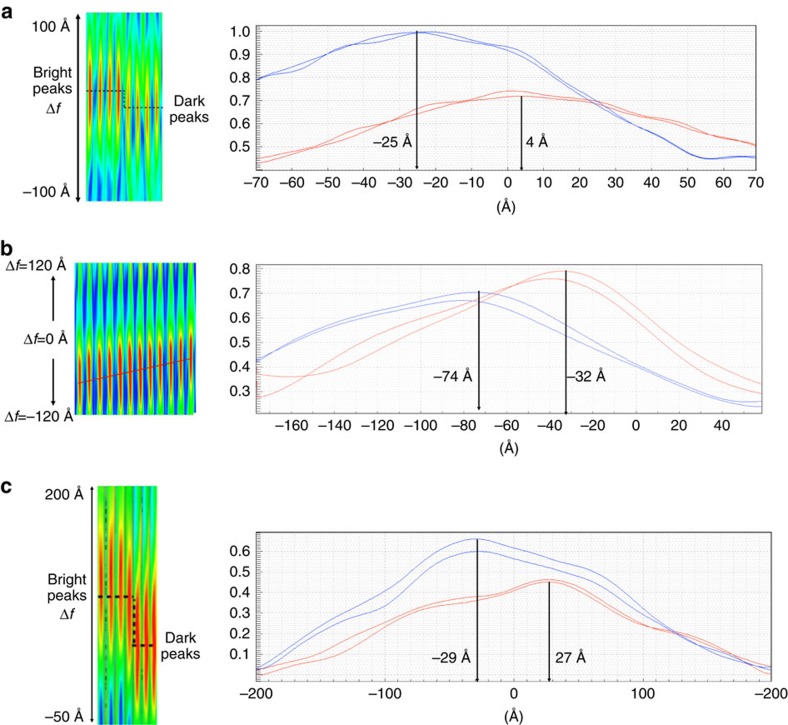
Intensity of the column wave as function of the defocussing distance. (**a**) Cross-section of propagated intensity (ellipsoid shape, left of panel) and intensity profiles (right of panel) from dark (blue) and bright (red) columns of modulus of Ge exit wave (Fig. 2a). (**b**) Cross-section of propagated intensity (ellipsoid shape) and intensity profiles from the centre (blue dot) to the edge (red dot) columns of the modulus of Au exit wave (Fig. 2b). (**c**) Cross-section of propagated intensity (ellipsoid shape) and intensity profiles from dark (blue) and bright (red) columns of modulus of MgO exit wave (Fig. 2c). In three cases, two columns at two different positions are analysed. The position of the atom locates at the maximum (indicated), which can be determined with a precision of the order of 0.1 nm.

We back propagate the exit wave function numerically by convolution with an inverse defocus operator. In this process, every spherical wave is refocused backwards into its point of origin where the intensity is maximal. This position can be obtained by monitoring the intensity of the back-propagated wave as a function of the propagated distance. The exit wave function was propagated (defocused) in both positive and negative directions, and for each column the intensity at every focus positions was recorded and forms a 3D (*x*, *y*, Δ*f*) intensity stack along the focus axis. We applied this to the cases of Ge [110], MgO [100] and Au [110]. Some cross-sections of defocused intensity stacks are displayed in Fig. 7a–c, respectively. All local column intensities of the waves show an elongated ellipsoidal shape (left in Fig. 7). The intensity profile (right in Fig. 7) along the centre of the ellipsoid are also given to show the difference in ‘*z*' height of the exit surface. However, the ellipsoid always exhibits a ‘flat' intensity maximum, which limits the depth precision.

In a second step, we improved this precision by the ‘Big-Bang' procedure^18^, which basically acts as follows. Because of the electron channelling, the exit wave function is sharply peaked at the centre of every column. When this wave propagates in free space towards the plane of observation, these peaks acts as point sources of spherical waves. It was recently outlined^23^ theoretically how the position of the exit plane for each atomic column in 3D can be determined very accurately from fitting of the intersection of this spherical wave in the plane of observation. Here, the three sets of experimental data are refined in this manner, yielding a precision of about 0.1 nm.

**Figure 8:**
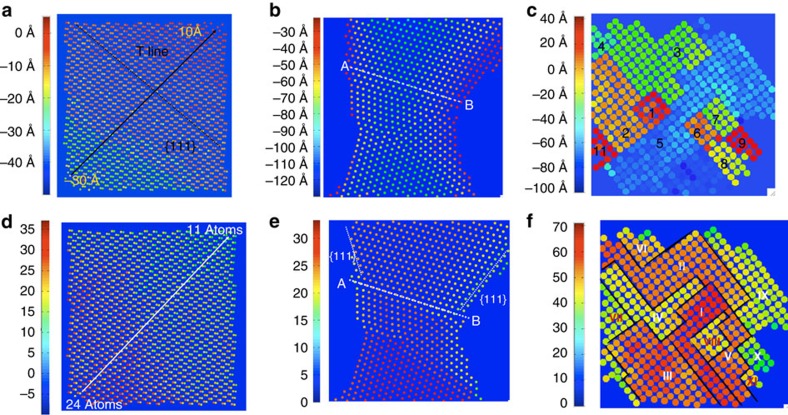
Focus maps and mass maps. (**a**) Focus map for Ge. (**b**) Focus map for Au bridge. (**c**) Focus map for MgO, which shows focus patches that correspond to the peaks indicated in the histogram (fig. 3c). (**d**) Mass map for Ge. (**e**) Mass map for Au bridge. (**f**) Mass map for MgO. The humps indicated in the mass histogram of MgO (fig. 3c) corresponds to the mass patches.

Figure 8 shows the focus maps that are obtained by correcting the focus in every atomic column and the corresponding focus histograms are shown in Fig. 3a–c. For Ge [110], we observe a focus gradient across the image from bottom left (∼−30 Å) to the top right (∼10 Å). In the focus map of Au, the focus value along the line A–B line shows a difference of ∼26–30 Å between the edge and centre parts. The focus map of the MgO exhibits a number of flat focus patches numbered from 1 to 11. This histogram is discrete at the Angstrom level, which suggests that the addition of single atoms to a column can be measured by a related change of focus.

**Figure 9:**
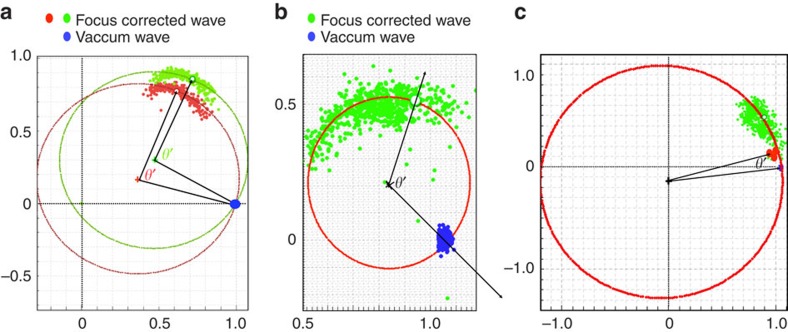
Plot of the mass circle and the phase of *θ*′. (**a**) Ge. (**b**) Au bridge. (**c**) MgO. The green dots and red dots are FCWs , and the blue dots are the vacuum wave. The green and red dots in Ge case are the FCW from right and left columns of the dumbbell pairs. The red dots in the MgO case are the FCW from the edge.

Thereafter, the experimental exit wave function of every column is then back propagated column so as to be used as the wave ψ for a determination of the mass of the corresponding columns. Next, another wave *ϕ* is determined in the ‘valley' between the atomic columns. Using the valley wave *ϕ*, the FCW ψ can then be ‘normalized' as follows.


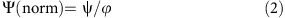


where Ψ (norm) is the normalized FCW so as to corrected by the mean inner potential of the crystal. Figure 9 shows the Argand plots for the cases of Ge [110], MgO [100] and Au [110] after focus correction, which reduces the data scatter significantly so that the expected circular arc can be recognized. The position of the point along this circular curve is now only a function of the column mass.

### Determination of the column mass

The last step then consists in determining the column mass for every individual column with respect to the vacuum wave. The green dots and the blue dots in the Fig. 9 are the FCWs and the vacuum waves, respectively, which are fitted with a ‘mass circle'. The red and green dots in the Fig. 9a are the FCWs of Ge. The two branches correspond with the left and right columns of the dumbbell and this difference is caused by sample tilt. The red dots in the Fig. 9c are the FCW functions of single MgO molecules from the edge of the crystal.

From the channelling theory, the projected mass of a column is proportional to the angle *θ*' measured from the centre of a mass circle. The radius of the mass circle depends on the electron dose rate and other damping functions as described in the main text. As the exit wave functions can only be determined apart from a constant phase offset, we use the vacuum wave to define (1,0). The column mass is then given by the difference between the phase of Ψ (norm) and the vacuum wave. This procedure applies to MgO and Au where vacuum values are visible in the images but not to the case of the Ge where the material fills the entire field of view (Fig. 2a). In that case, we have set the vacuum wave to the theoretical position (1,0). The green and red circles in Fig. 9a–c are the fitted mass circles for Ge, MgO and Au, respectively. The mass associated with a column can be calibrated in terms of number of atoms by


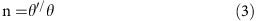


where *θ* is the phase change/atom given in Table 1 and Methods.

Concerning an absolute calibration of the phase change per atom in a column, which is commonly influenced by electron beam-induced and thermal vibrations, nature offers its own proper yardstick (M-II). Figure 8d–f show the mass maps of the investigated samples (mass histograms are depicted in Fig. 3d–f). Discrete values are observed, because the contrast contribution from scattering at single atoms can be isolated in high-performance microscopes. The number of atoms in each detected column is used to form the histograms and the maps. The Ge mass map shows a mass gradient of ∼24 atoms in the bottom left corner towards the top right corner ∼11 atoms along the diagonal direction. A mass profile along line A–B of the Au[110] sample reveals a difference of seven to eight atoms between the edge and centre of the sample. The mass map of the MgO shows the number of MgO molecules in each patch # I to # X. Along [001] projection, the Mg and O atoms alternate in each column. In our analysis, the Mg and O atoms are treated as a pair of atoms and the number of MgO molecules are deduced from the mass circle in Fig. 9c.

Based on the focus and mass maps shown in the Fig. 8, we built the 3D tomography of Ge, MgO and Au samples that are shown in Fig. 4. It is worth mentioning that with our present method we cannot unambiguously determine the termination of the {100} surfaces with either Mg or O atoms from only one projection even though patches of relevant checkerboard patterns exist in the images and a projection along another crystallographic orientation that shows the Mg and O alternating layers would be helpful.

### The phase change per atom *θ* (rad per atom)

For an absolute calibration of the phase change per atom in a column, which is commonly influenced by electron beam-induced and thermal vibrations, nature offers its own proper yardstick.

The phase change per atom *θ* for Ge, Au and MgO is calculated along [001] crystallographic orientation with multislice simulation using MacTempas (totalresolution.com) for different thicknesses. The imaging parameters are those for the 300-keV TEAM0.5 microscope with *g*_max_ set at 2 Å^−1^. The Debye–Waller factor is 0.5 Å^2^. The simulation were carried out for thickness up to eight atoms with increments of one atom (or molecule for MgO). The peak-to-valley phase values were measured from electron exit wave functions that were reconstructed from focus series of simulated images for the different thicknesses. Peak-to-valley phase values *θ* (rad per atom) are as follows: 0.16 for Ge (a.m.u.=72.6), 0.09 for MgO (a.m.u.=40.3) and 0.4 for Au (a.m.u.=197). It was reported^26^ that the phase/atom scales with the atomic number *Z*^2/3^. A comparison with experimental values is given in Table 1, where it is seen that deviations from the expectations increase with increasing dose rate. Part of the deviation comes from the potential softening by e-beam excitations and part from an imperfect recovery of the background phase.

## Additional information

**How to cite this article**: Chen, F.-R. *et al.* In-line three-dimensional holography of nanocrystalline objects at atomic resolution. *Nat. Commun.* 7:10603 doi: 10.1038/ncomms10603 (2016).

## Figures and Tables

**Table 1:** Phase change/atom, accuracy in the mass phase and dose rate for Ge, Au and MgO.

**Material/*****Z*** **(a.m.u.)**	**Peak to valley phase change/atom (rad)(experimental measurement)**	**Phase change per atom (rad) (calculated)**	**Thickness accuracy (nm), 2*****σ***	**Phase accuracy (rad), 2*****σ***	Dose rate * 10^3^ (e/A^−2^s^−1^)
Germanium /32 (72.63)	0.11 Extrapolated by *Z*^2/3^	0.16	0.12	0.07	15
gold/79 (179)	0.21	0.40	0.24	0.13	46
Magnesium oxide/20 (40)	0.08	0.09	0.22	0.04	1.3
